# t-PA/PAI-1 complex expression correlates with the severity in hantaan virus-induced hemorrhagic fever with renal syndrome

**DOI:** 10.3389/fcimb.2026.1849586

**Published:** 2026-07-08

**Authors:** Han-Dong Zhao, Fei-Yu Li, Hong-Bo Qian, Pei-Long Wang, Kang-Xiao Ma, Hong-Li Liu

**Affiliations:** 1 Central Laboratory of Virology, Shaanxi Provincial Hospital of Infectious Diseases, The Xi’an Eighth Hospital, Xi’an, China; 2 Clinical Laboratory Center, Shaanxi Provincial Hospital of Infectious Diseases, The Xi’an Eighth Hospital, Xi’an, China; 3 Shaanxi Provincial Clinical Medical Research Center of Infectious Diseases, Shaanxi Provincial Hospital of Infectious Diseases, The Xi’an Eighth Hospital, Xi’an, China; 4 Section of Science and Education, Shaanxi Provincial Hospital of Infectious Diseases, The Xi’an Eighth Hospital, Xi’an, China; 5 Clinical Laboratory Center, Xi’an People’s Hospital (Xi’an Fourth Hospital), Affiliated Hospital of Northwest University, Xi’an, China

**Keywords:** disease monitoring, evaluation, hantaan virus, HFRS, t-PAIC

## Abstract

**Objectives:**

This study aimed to investigate t-PAIC concentration changes across severity levels in HFRS patients and evaluate its value in predicting disease severity, providing a potential biomarker for HFRS severity assessment and monitoring.

**Methods:**

t-PAIC levels were measured by chemiluminescence immunoassay in 156 HFRS patients and 37 healthy controls. Routine laboratory tests assessed WBC, PLT, Cr, UA, urea, HDL, ALB, APTT, and PT. HFRS-specific antibodies were detected using the colloidal gold method. Spearman correlation analysis evaluated associations between t-PAIC and clinical indices. Ordinal logistic regression identified risk factors linked to disease severity. ROC curve analysis assessed the predictive performance of t-PAIC for HFRS severity.

**Results:**

t-PAIC levels progressively increased with HFRS severity, peaking in critically ill patients. t-PAIC showed significant positive correlations with leukocyte count (*r* = 0.6082; *p* < 0.01), Cr (*r* = 0.3567; *p* < 0.01), urea (*r* = 0.4376; *p* < 0.01), UA (*r* = 0.3201; *p* < 0.01), APTT (*r* = 0.6182; *p* < 0.01), and PT (*r* = 0.2997; *p* < 0.01), and significant inverse correlations with PLT (*r* = −0.6177; *p* < 0.01), HDL (*r* = −0.3074; *p* < 0.01), and ALB (*r* = −0.4790; *p* < 0.01). ROC analysis revealed t-PAIC had good predictive value for disease severity (AUC = 0.796, 95% *CI*: 0.724–0.867, *p* < 0.001).

**Conclusions:**

t-PAIC levels closely reflect HFRS severity and associated clinical features, including renal dysfunction and coagulation abnormalities, suggesting its potential as a biomarker for monitoring disease status.

## Introduction

Hemorrhagic fever with renal syndrome (HFRS), an ancient yet recurrent infectious disease, continues to pose a significant threat to public health and has garnered increasing global attention. The earliest recorded description of this disease dates back to a Chinese medical text from 960 AD (Noh et al., 2019). In modern times, HFRS was first reported in South Korea in 1951 during the Korean War. However, the causative agent, Hantaan virus (HTNV), remained unidentified until 1976 and was subsequently confirmed by researchers in 1978 (Smadel, 1953; French et al., 1981). HTNV is a zoonotic, negative-sense RNA virus belonging to the family *Elliovirales* within the order *Bunyaviricetes* (Walker et al., 2019; (ICTV), 2026). The striped field mouse (*Apodemus agrarius*) serves as the natural reservoir host of this virus, typically exhibiting an asymptomatic infection. Humans, however, can become infected through inhalation of aerosols contaminated with excreta—such as saliva, urine, and feces—from infected rodents. Initial symptoms are often nonspecific and resemble influenza, including fever and headache. These may progress to more severe manifestations such as hypotension, thrombocytopenia, and renal impairment. In severe cases, patients may develop multiple organ dysfunction syndrome (MODS) (Avsic-Zupanc et al., 2019; Vaheri et al., 2013a; Muranyi et al., 2005). Furthermore, comorbidities affecting the renal, cardiovascular, and endocrine systems have been reported following HTNV infection (Vaheri et al., 2013b). HFRS is endemic across Europe and Asia. China bears a substantial disease burden, accounting for approximately 90% of global HFRS cases. Between 1950 and 2014, a total of 1,625,002 HFRS cases and 46,968 HFRS-related deaths were documented in China. In 2021 alone, 2,657 HFRS cases were reported in Shaanxi Province, coinciding with the ongoing period of the COVID-19 pandemic (Yan et al., 2007; Wei et al., 2021). With increasing insights into the pathogenesis of this disorder, capillary and small vessel endothelial cells are now recognized as the primary targets of hantaviruses, and enhanced vascular permeability represents a hallmark feature of the pathological alterations observed in HFRS (Noack et al., 2020). Although integrated interventions—including rodent control, environmental management, and vaccination—have substantially reduced the incidence of HFRS, the persistent or re-emergent transmission in endemic regions poses ongoing challenges for disease surveillance (Liu et al., 2019). Therefore, identifying a reliable clinical parameter to reflect the disease status of HFRS patients is of critical importance, as it would also facilitate severity assessment and prognosis prediction in clinical practice.

The t-PA/PAI-1 complex (t-PAIC) consists of tissue plasminogen activator (tPA) and plasminogen activator inhibitor 1 (PAI-1). The former is a 68-kDa serine protease that was initially purified and characterized in human circulation and later in the uterus in 1979, while the latter is a single-chain 45-kDa glycoprotein composed of 379 or 381 amino acids, belonging to the serine proteinase inhibitor superfamily, and was first reported in 1986 (Suzuki et al., 2009; Yasar Yildiz et al., 2014). PAI-1 can be produced by various cell types and tissues, including vascular smooth muscle cells, adipocytes, splenocytes, and hepatocytes (Tsantes et al., 2008). Notably, both PAI-1 and tPA are secreted by endothelial cells—the primary target of HTNV infection, as previously mentioned—suggesting a potential role of t-PAIC in the pathogenesis of HFRS (Noack et al., 2020; Suzuki et al., 2009; Tsantes et al., 2008). Indeed, recent studies have demonstrated that t-PAIC serves as an important biomarker in venous thrombosis, with levels markedly elevated prior to thrombotic events. Moreover, plasma concentrations of this complex have been significantly associated with the risk of myocardial infarction in both men and women (Nordenhem et al., 2005). In addition, t-PAIC levels are considered an independent predictor of all-cause and cardiovascular mortality in patients with heart failure with preserved ejection fraction (Winter et al., 2017). Moreover, fluctuations in this complex have been closely associated with postoperative bleeding following cardiac surgery; furthermore, t-PAIC has been proposed as a potential biomarker for the early diagnosis, assessment, and prognosis of sepsis-induced coagulopathy (Ozolina et al., 2012; Li et al., 2023). Given that HFRS patients exhibit an increased risk of coagulation disorders—evidenced by a higher incidence of disseminated intravascular coagulation (DIC), acute myocardial infarction (AMI), and stroke—and considering the critical roles of t-PA and PAI-1 in regulating plasmin within the fibrinolytic system (Suzuki et al., 2009; Tsantes et al., 2008; Chen et al., 2024), the association between t-PAIC levels and the severity of Hantaan virus-induced HFRS remains to be fully elucidated.

Accordingly, in this study, we examined t-PAIC levels across different stages of HFRS; analyzed the associations between this complex and markers of renal function, coagulation, and inflammation; and evaluated the correlation between fluctuations in t-PAIC concentrations and the clinical severity of HFRS. Furthermore, the predictive value of t-PAIC for disease severity was assessed using receiver operating characteristic (ROC) curve analysis.

## 2 Materials and methods

### 2.1 Study population

**Figure 1:**
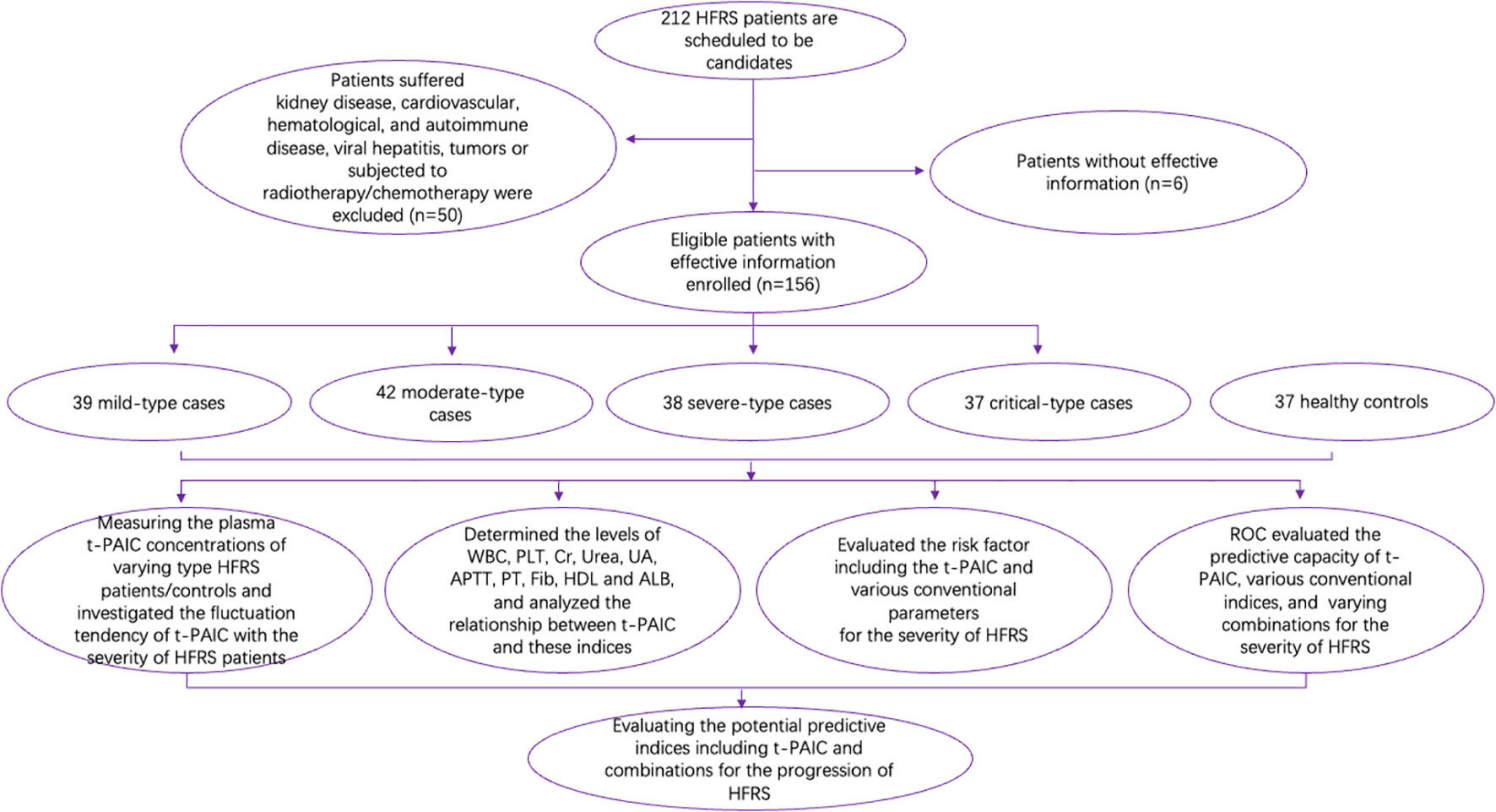
Flowchart of the study. t-PAIC, t-PA/PAI-1 complex; HFRS, hemorrhagic fever with renal syndrome; ROC, receiver operating characteristics curve; WBC, white blood cell; PLT, Platelets; Cr, Creatinine; UA, Uric acid; APTT, Activated partial thromboplastin time; PT, Prothrombin time; HDL, High-density lipoprotein; ALB, Albumin.

This study is a cross-sectional, single-center research that initially recruited 212 HFRS patients who were hospitalized at the Department of Infectious Diseases of Shaanxi Provincial Hospital of Infectious Diseases (The Xi’an Eighth Hospital) from November 2023 to May 2025 as candidates, and finally 156 patients who meet the criteria described below were enrolled with a correspondence 37 healthy individuals as controls (**Figure 1**). Specifically, patients were recruited according to the HFRS criteria for clinical diagnosis and classification, released by the National Health Commission of China which included (China, 2008): (i) epidemiological history that travel or living in an epidemic region, or have a record of direct or indirect contacts with rodent excreta, (ii) clinical manifestations including fever, fatigue, nausea, vomiting, hemorrhage, hypotensive shock, or kidney impairment. (iii) positive serum-specific antibodies for Hantaan virus. Meanwhile, the patients were excluded based on the criteria as follows: (i) viral hepatitis or other viral infection, (ii) autoimmune diseases, (iii) hematological diseases, (iv) patients who suffered tumors and were subjected to radiotherapy and chemotherapy. The clinical information and laboratory testing data were collected and analyzed anonymously, and informed consent was provided by the participants. All procedures were performed as the ethical standards outlined in the 1964 Helsinki Declaration and its subsequent amendments or comparable ethical standards and were approved by the Institutional Review Board of Shaanxi Provincial Hospital of Infectious Disease.

### 2.2 Definition

The severity of HFRS patients was categorized into four subgroups based on the clinical manifestations and disease progression: mild cases (n=39) were characterized by renal injury without significant hypotension or oliguria; moderate cases (n=42) presented with hypotension, skin and mucous membrane hemorrhages, bulbar conjunctival congestion, and acute kidney injury (AKI) accompanied by oliguria; severe cases (n=38) exhibited marked uremia, persistent hypotension, skin and mucous membrane bleeding, bulbar conjunctival edema, peritoneal or pleural effusion, and AKI defined by a daily urine output of 50–500 mL for up to 5 days, or a reduction in urine output to less than 100 mL per day for no more than 2 days; critical cases (n=37) demonstrated one or more life-threatening complications, including refractory shock lasting at least 2 days, severe AKI with a daily urine output of 50–500 mL for up to 5 days or less than 100 mL per day for up to 2 days, severe secondary infection or heart failure, visceral hemorrhage, or pulmonary and cerebral edema. In addition, the clinical course was also divided into the acute phase—which encompasses the febrile, hypotensive, and oliguric stages—and the convalescent phase—which includes the diuretic and convalescent stages—based on the criteria outlined above by the National Health Commission of China (China, 2008).

### 2.3 Examination of routine laboratory parameters

Peripheral blood was collected from the venous circulation of both patients and healthy individuals, followed by centrifugation at 537 relative centrifugal force (g) (TD4A, Changsha Yingtai Instrument, China) for 10 minutes at 4 °C. Ten laboratory parameters were measured using automated analyzers as per standard protocols (Mindray SAL9000, Mindray BC6900, Mindray ExC810; Mindray Medical, China), including white blood cell (WBC) count, platelet (PLT) count, activated partial thromboplastin time (APTT), prothrombin time (PT), fibrinogen, creatinine (Cr), urea, uric acid (UA), high-density lipoprotein (HDL), and albumin (ALB).

### 2.4 Examination of HTNV antibody

The colloidal gold method was employed to detect antibody titers specific for HTNV according to the manufacturer’s instructions (Bosheng Biotechnology, China). First, 2 µL of EDTA-K2 anticoagulated plasma was added to 100 µL of sample dilution solution; subsequently, 70 µL of the mixture was transferred onto the test device and incubated for 20 min as per the prescribed protocol. Results were interpreted within 15 min. The assay kits exhibited a sensitivity of 96.71% and a specificity of 98.72%.

### 2.5 Examination of t-PA/PAI-1 complex

Venous blood samples were collected from both HFRS patients and healthy individuals and transferred into sodium citrate anticoagulant tubes, followed by a 10-minute incubation at 37 °C. Subsequently, plasma was separated by centrifugation at 1209 relative centrifugal force (g) (TD4A, Changsha Yingtai Instrument, China) for 10 minutes. The concentrations of the t-PA/PAI-1 complex were determined using the chemiluminescence immunoassay (CLIA) method on an automated analyzer according to the manufacturer’s instructions (Wondfo I2900, Wondfo Bio, China). In brief, 10 µL of plasma and 50 µL of magnetic bead working solution were added to the reaction vessel and incubated for 5 minutes at 37 °C, then washed. Next, 50 µL of alkaline phosphatase (ALP)-labeled working solution was added and incubated at 37 °C for another 5 minutes. Luminescence signals emitted from the double-antibody sandwich complex—comprising magnetic beads coated with antibody, the t-PA/PAI-1 complex, and ALP-labeled antibody—were subsequently measured at a wavelength of 470 nm.

### 2.6 Statistical analysis

Figures were generated using Origin 2021 software (OriginLab Corporation, USA), while tables were prepared using Excel 2021 (Microsoft Corporation, USA). Statistical analyses were performed with GraphPad Prism 8.0 software (GraphPad Software, LLC, USA). Statistics values are presented as medians with interquartile ranges (IQR) and were analyzed using the Kruskal-Wallis H test, as appropriate. Spearman’s correlation analysis was employed to evaluate the association between the t-PA/PAI-1 complex and multiple conventional laboratory parameters. The risk factors for disease severity were assessed through ordinal logistic regression analysis, and the predictive performance of the t-PAIC for the severity of HFRS was evaluated using receiver operating characteristic (ROC) curve analysis, with results reported as the area under the ROC curve (AUC), the combined models were constructed using logistic regression-derived probabilities. A *P* value < 0.05 was considered statistically significant.

## 3 Results

### 3.1 General information and characteristics of HFRS patients

Although 212 laboratory-confirmed HFRS patients were initially considered eligible for this study, individuals with concomitant viral hepatitis or other viral infections, autoimmune disorders, hematological diseases, tumors, or those who had undergone radiotherapy or chemotherapy were excluded based on the enrollment criteria. As a result, a total of 156 HFRS patients were enrolled and classified according to the guidelines issued by the National Health Commission of China. The patient distribution comprised 39 mild cases, 42 moderate cases, 38 severe cases, and 37 critical cases. As shown in **Table 1**, male patients predominated across all disease severity groups, and hospitalization duration progressively increased with the worsening of clinical condition. Furthermore, a consistent upward trend was observed in several conventional laboratory parameters, including WBC, Cr, urea, uric acid UA, and APTT. In contrast, indices such as PLT, HDL, and ALB exhibited a marked decline that correlated with disease progression. Notably, significantly reduced Fib levels were observed only in critical-type patients, with no significant reduction detected in mild, moderate, or severe cases.

**Table 1:** Baseline demographics and clinical characteristics of patients with hemorrhagic fever with renal syndrome.

Classification and recruited number of study population.						
	Mild group	Moderate group	Severe group	Critical group	Controls group	Normal range
	(n=39)	(n=42)	(n=38)	(n=37)	(n=37)	
Gender						
Female, n (%)	6 (15.38)	7 (16.67)	5 (13.16)	5 (13.51)	9 (24.32)	–
Male, n (%)	33 (84.62)	35 (83.33)	33 (86.84)	32 (86.49)	28 (75.68)	–
Age, y	30 (24-38)	42.5 (29.75-52.25)	42 (34.25-47.25)	48 (35.5-55.5)	31 (25-36)	–
Hospitalization, days	8 (7-11)	11.5 (9.75-16.25)	16 (12.75-18)	20 (15.5-22.5)		–
WBC, ×109/L	8.21 (6.04-10.44)	11.98 (9.92-14.11)	14.55 (12.35-19.23)	22.73 (14.11-33.18)	8.36 (6.85-9.59)	4–10
PLT, ×109/L	104 (70-144)	60 (47.75-78.25)	41.5 (28.5-54.25)	19 (12.5-39)	128 (103-156)	100–300
Cr, μmol/L	96.7 (77-127.6)	227 (134.93-286.98)	465.55 (319.88-556.58)	404.7 (304.25-548.9)	94.6 (83.3-109.8)	70 – 115
Urea, mmol/L	6.24 (4.74-8.39)	13.08 (9.03-16.77)	19.75 (15-25.06)	22.16 (19.66-29.08)	4.25 (3.47-4.89)	1.7 – 8.3
UA, μmol/L	399.2 (297-458.7)	475.15 (446.75-530.08)	527.5 (464.05-591.55)	557.6 (470.75-725.65)	237 (189.25-286.65)	202– 416
APTT, sec	33.3 (29.73-36.47)	39.735 (33.44-46)	48.28 (37.5-59.48)	74.82 (45.36-97.46)	25.63 (23.84-32.96)	24 – 38
PT, sec	13.31 (12.05-13.79)	13.10 (12.16-14.12)	13.22 (12.45-14.32)	14.17 (12.67-16.8)	12.91 (12.31-14.24)	10 – 15
Fib, g/L	3.05 (2.60-3.44)	3.14 (2.67-3.64)	3.05 (2.56-4.03)	1.96 (1.75-2.73)	2.42 (2.07-2.91)	2 – 4
HDL, mmol/L	0.85 (0.73-0.99)	0.74 (0.58-0.85)	0.69 (0.49-0.9)	0.61 (0.44-0.73)	0.92 (0.82-1.05)	0.83-1.96
ALB, g/L	37 (35-40)	33.75 (31.2-35.55)	32.2 (28.2-34.7)	28.65 (26.7-30.5)	40.2 (36.3-45.2)	35 – 55

WBC, white blood cell; PLT, Platelets; Cr, Creatinine; UA, Uric acid; APTT, Activated partial thromboplastin time; PT, Prothrombin time; Fib, Fibrinogen; HDL, High-density lipoprotein; ALB, Albumin.

The gender information are presented as n (%), while the other data are presented as Medians (Q1–Q3).

### 3.2 Association between t-PAIC fluctuation and the severity of HFRS

**Figure 2:**
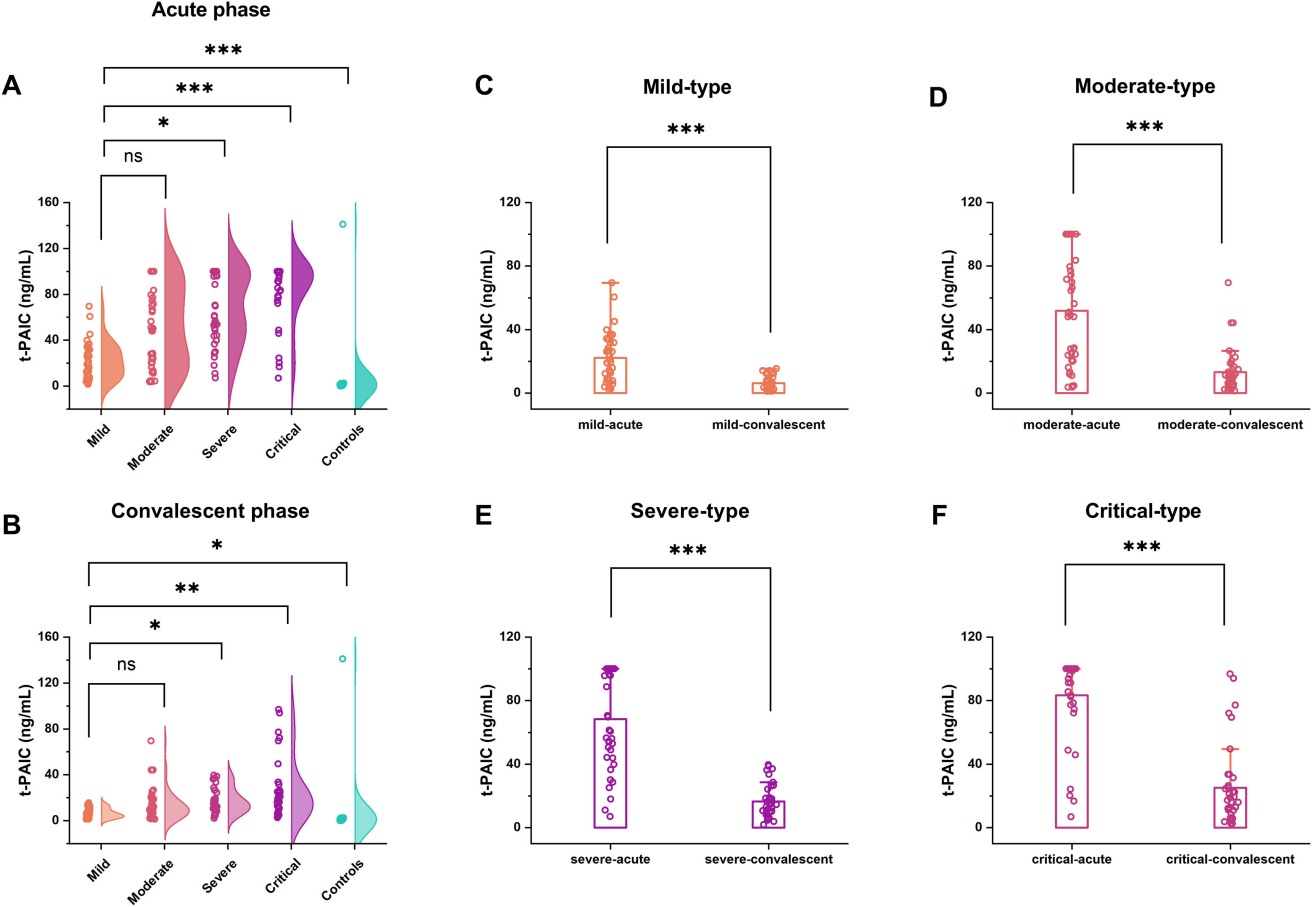
The fluctuation tendency of t-PAIC in patients suffered hemorrhagic fever with renal syndrome. The levels of t-PAIC in patients with HFRS of varying disease severity were assessed using chemiluminescence immunoassay. A significant increase in t-PAIC levels was observed from mild to critical cases during the acute phase [*p < 0.05, **(A)**], although no statistically significant difference was detected between mild and moderate cases [ns; *p*>0.05, **(B)**]. Furthermore, a marked reduction in t-PAIC levels was evident when comparing the acute phase to the convalescent phase within each disease severity group [***p < 0.001, **(C–F)**]. Notably, t-PAIC levels in all patient groups during the convalescent phase remained significantly higher than those in the control group under the same conditions [*p < 0.05, **(B)**]. Healthy individuals were enrolled as controls and had no history of Hantaan virus infection or exposure to the acute or convalescent phases of HFRS. A significant difference can also be found between the mild and critical type HFRS patients in the same scenario (**p < 0.01).

The concentrations of t-PAIC were measured across different subgroups to explore the association between t-PAIC level changes and the progression of HFRS. As illustrated in **Figure 2**, a significant elevation in t-PAIC expression was observed with the worsening severity of HFRS, with the highest levels detected in patients with critical-type HFRS during the acute phase (**Figure 2A**). Moreover, a clear distinction in t-PAIC levels was evident between the control group and patients with mild-type HFRS, although no significant difference was observed between the mild and moderate subgroups during the same phase. Furthermore, a marked decline in t-PAIC levels from the acute phase to the convalescent phase was noted across all patient groups. The differences in t-PAIC concentrations among patients with mild, moderate, and severe HFRS became less pronounced during the convalescent phase. Nevertheless, even in the convalescent phase, t-PAIC levels in patients with mild-type HFRS remained significantly higher than those in the control group, despite their relatively mild clinical course.

### 3.3 Correlation of the t-PAIC with the conventional indices

**Figure 3:**
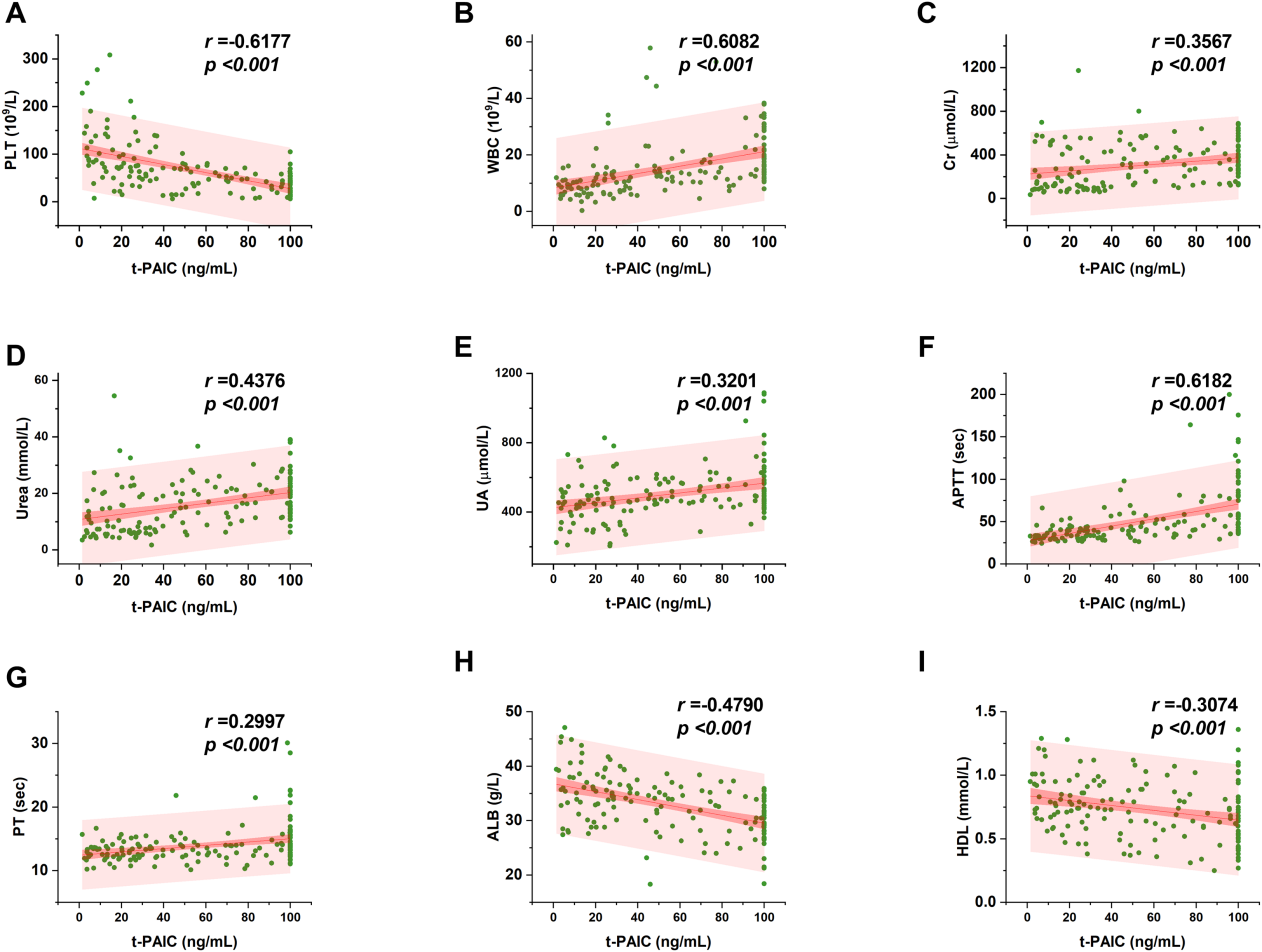
Correlation of the t-PAIC with routine parameters. A significant negative correlation was observed between t-PAIC and PLT concentrations in patients with HFRS [*p* < 0.001, *r* = -0.6177, **(A)**]. Similarly, negative correlations were also detected between t-PAIC and both ALB, as well as HDL [*p* < 0.001, *r* = -0.4790; *p* < 0.001, *r* = -0.3074, **(H, I)**]. In contrast, the fluctuation pattern of t-PAIC showed a positive correlation with changes in WBC levels [*p* < 0.001, *r* = 0.6082, **(B)**]. This positive association was also evident between t-PAIC and other routine laboratory parameters, including Cr, urea, UA, APTT, and PT [*p* < 0.001, *r* = 0.3567; *p* < 0.001, *r* = 0.4376; *p* < 0.001, *r* = 0.3201; *p* < 0.001, *r* = 0.6182; *p* < 0.001, *r* = 0.2997, **(C–G)**].

Considering that HFRS patients exhibit an elevated coagulation risk—manifested by a higher incidence of DIC—with severe cases potentially progressing to MODS, and given that increased microvascular permeability in endothelium-containing organs, including the kidney, has been well documented, the correlation between t-PAIC and routine clinical parameters such as PLT, WBC, Cr, urea, UA, APTT, PT, ALB, and HDL was investigated. Spearman correlation analysis revealed a significant negative correlation between t-PAIC and PLT (**Figure 3A**; *p* < 0.01, *r* = -0.6177). Similarly, significant negative correlations were also observed between t-PAIC and both ALB and HDL (**Figures 3H, I**; *p* < 0.01, *r* = -0.4790; *p* < 0.01, *r* = -0.3074, respectively). In contrast, t-PAIC levels showed positive correlations with changes in WBC, Cr, urea, UA, APTT, and PT (**Figures 3B–G**; all *p* < 0.01). Notably, the strength of these correlations varied. The strongest inverse relationship was observed between t-PAIC and PLT, followed by a moderate inverse correlation with ALB, while the association with HDL exhibited the weakest negative correlation. Among the positive correlations, the association between t-PAIC and APTT was the most pronounced, whereas the correlation between t-PAIC and PT was the least robust.

### 3.4 Evaluation of severity risk factor in HFRS

Ordinal logistic regression analysis was conducted to investigate risk factors associated with disease severity in patients with HFRS. As shown in **Table 2**, changes in the levels of t-PAIC, WBC, PLT, Urea, and UA were identified as statistically significant risk factors. Specifically, elevated concentrations of t-PAIC, WBC, Urea, and UA were positively associated with increased disease severity, whereas a decline in PLT levels was linked to more severe manifestations of HFRS. Furthermore, decreasing levels of HDL, ALB, APTT, and Fib exhibited a potential influence on disease progression, similar to that observed for PLT, although these associations did not reach statistical significance.

**Table 2:** Ordinal logistic regression analysis of risk factors for disease severity in patients with HFRS.

Variables	Estimated value	p-value	OR	(95% CI)
				(lower - upper)
t-PAIC	0.018	0.011	1.018	(1.004 -1.033)
WBC	0.062	0.032	1.064	(1.005 -1.126)
PLT	-0.02	0.006	0.980	(0.966 - 0.994)
HDL	-0.781	0.394	0.458	(0.076 -2.763)
ALB	-0.087	0.394	0.917	(0.823 - 1.022)
Cr	0.003	0.061	1.003	(1.00 - 1.005)
Urea	0.119	0.001	1.126	(1.053 - 1.205)
UA	0.007	0.001	1.007	(1.003 - 1.011)
APTT	-0.005	0.542	0.995	(0.979 - 1.011)
PT	0.085	0.324	1.089	(0.920 - 1.289)
Fib	-0.135	0.570	0.874	(0.549 - 1.392)

t-PAIC, t-PA/PAI-1 complex; WBC, White blood cells; PLT, Platelets; HDL, High-density lipoprotein; ALB, Albumin; Cr, Creatinine; UA, Uric acid; APTT, Activated partial thromboplastin time; PT, Prothrombin time; Fib, Fibrinogen.

OR, The OR value reflects the relative risk of an outcome occurring in the presence or absence of exposure; CI, Confidence interval.

### 3.5 Evaluation of predictive efficacy for the severity of HFRS

**Figure 4:**
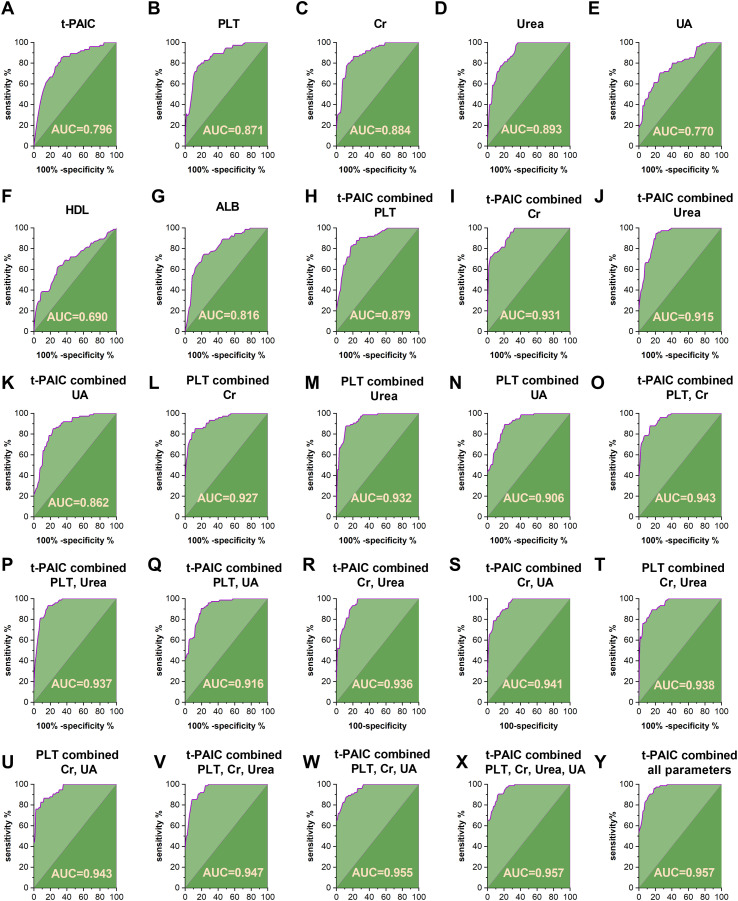
Predictive capacity of t-PAIC, conventional indices, and varying combinations. ROC analysis demonstrated that t-PAIC exhibited significant predictive capacity for the severity of HFRS, with an AUC of 0.796 [*p* < 0.001, **(A)**]. A similar significant predictive performance was also observed for conventional indices, including PLT, Cr, urea, ALB, UA, and HDL [*p* < 0.001, **(B–G)**], although the AUC values for the latter two parameters (ALB and HDL) were lower than those of the other indices [*p* < 0.001, **(E, F)**]. The predictive performance improved progressively with an increasing number of combined indices [*p* < 0.001, **(H–W)**]. The highest AUC values were achieved when t-PAIC was combined with PLT, Cr, urea, and UA, as well as when t-PAIC was combined with all conventional indices (*p* < 0.001, X–Y).

The predictive performance of t-PAIC, various conventional parameters, and the combination of t-PAIC with these indices was evaluated using ROC curve analysis. As shown in **Figure 4** and **Table 3**, ROC curve analysis revealed a notable predictive ability for several parameters, all of which exhibited an AUC greater than 0.70, including t-PAIC, PLT, Cr, urea, UA, and ALB (**Figures 4A–E, G**). In contrast, HDL showed slightly lower discriminative power (**Figure 4F**; AUC = 0.690, 95% CI: 0.606–0.773, *p* < 0.01). Notably, although the AUC values for PLT, Cr, urea, and UA were higher than that of t-PAIC, t-PAIC demonstrated a superior balance between sensitivity and specificity, highlighting its enhanced value in predicting the severity of HFRS. Moreover, hemolysis and erythrocyte fragments can significantly interfere with the laboratory measurement of PLT, Cr, and UA, potentially leading to misinterpretation and adversely affecting clinical decision-making during patient management. In addition, combining t-PAIC with one or more conventional markers further improved the predictive accuracy for HFRS severity (**Figures 4H–W**). Particularly, the combination of t-PAIC with PLT, Cr, urea, and UA achieved an AUC of 0.957 (**Figure 4X**; 95% *CI*: 0.930–0.983, *p* < 0.01), which is equivalent to the AUC obtained from the model incorporating t-PAIC with all parameters. Although the latter demonstrated higher sensitivity (**Figure 4Y**; **Table 3**), the former exhibited a more favorable balance between sensitivity and specificity, making it a particularly robust and clinically practical predictive model.

**Table 3:** Predictive performance of t-PAIC, conventional laboratory indices, and their combined use.

Variables	AUC (95% CI)	Sensitivity %	Specificity %	P-value
t-PAIC	0.796 (0.724 -0.867)	85.3333	66.6667	<0.001
PLT	0.871 (0.816 -0.926)	77.3333	83.9506	<0.001
Cr	0.884 (0.832 - 0.936)	86.6667	79.0123	<0.001
Urea	0.893 (0.843 -0.942)	100.0000	62.9630	<0.001
UA	0.770 (0.696 -0.843)	70.6667	74.0741	<0.001
HDL	0.690 (0.606 -0.773)	62.6667	70.3704	<0.001
ALB	0.816 (0.750 -0.883)	64.0000	87.6543	<0.001
t-PAIC combined PLT	0.879 (0.826 -0.932)	82.6667	81.4815	<0.001
t-PAIC combined Cr	0.931 (0.894 -0.968)	72.0000	96.2963	<0.001
t-PAIC combined Urea	0.915 (0.871 -0.958)	94.6667	79.0123	<0.001
t-PAIC combined UA	0.862 (0.804 -0.919)	85.3333	76.5432	<0.001
PLT combined Cr	0.927 (0.888-0.965)	85.3333	87.6543	<0.001
PLT combined Urea	0.932 (0.892 -0.971)	88.0000	87.6543	<0.001
PLT combined UA	0.906 (0.862 -0.950)	89.3333	79.0123	<0.001
t-PAIC combined PLT and Cr	0.943 (0.910 -0.975)	88.0000	86.4198	<0.001
t-PAIC combined PLT and Urea	0.937 (0.900 -0.974)	93.3333	82.7160	<0.001
t-PAIC combined PLT and UA	0.916 (0.874 -0.957)	90.6667	80.2469	<0.001
t-PAIC combined Cr and Urea	0.936 (0.900 -0.972)	100.0000	74.0741	<0.001
t-PAIC combined Cr and UA	0.941 (0.907 -0.974)	78.6667	92.5926	<0.001
PLT combined Cr and Urea	0.938 (0.902 -0.974)	89.3333	83.9506	<0.001
PLT combined Cr and UA	0.943 (0.910 -0.975)	86.6667	87.6543	<0.001
t-PAIC combined PLT, Cr and Urea	0.947 (0.916 -0.979)	85.3333	91.3580	<0.001
t-PAIC combined PLT, Cr and UA	0.955 (0.928 -0.982)	88.0000	87.6543	<0.001
t-PAIC combined PLT, Cr, Urea and UA	0.957 (0.930 -0.983)	90.6667	86.4198	<0.001
t-PAIC combined ALL	0.957 (0.930 -0.984)	96.0000	81.4815	<0.001

AUC, Area under the ROC curve; CI, Confidence interval; t-PAIC, t-PA/PAI-1 Complex; PLT, Platelets; Cr, Creatinine; UA, Uric acid; HDL, high-density lipoprotein; ALB, albumin.

## 4 Discussion

HFRS, a globally prevalent infectious disease caused by HTNV, exerts a significant impact on public health and socioeconomic conditions. Despite increasing efforts to prevent outbreaks and control the spread of this disorder, limitations in the availability and application of specific therapeutics, standardized treatment protocols, and widely accepted vaccines have led to the reemergence of HFRS in endemic regions (Sehgal et al., 2023). Consequently, the persistent emergence and widespread prevalence of this disease continue to pose challenges for monitoring and assessing disease severity in clinical settings, severely compromising the efficiency of patient management. These ongoing issues highlight the urgent need to identify a reliable biomarker for monitoring disease progression and evaluating clinical outcomes in HFRS patients.

In this study, we measured the concentrations of t-PAIC in patients with HFRS across varying degrees of disease severity. A marked elevation in t-PAIC levels was observed from mild to critical cases, although no significant difference was detected between mild and moderate patient groups. Notably, a clear distinction in t-PAIC levels emerged even between healthy controls and patients with mild HFRS, despite the relatively mild nature of HTNV infection in the latter. Moreover, t-PAIC levels during the acute phase of HFRS were significantly higher than those in the convalescent phase across all severity categories. Given that both t-PA and PAI-1—the constituent components of t-PAIC—are primarily secreted by endothelial cells and considering that the endothelium is recognized as the principal target of HTNV infection (Noack et al., 2020; Suzuki et al., 2009; Tsantes et al., 2008). The observed fluctuation in t-PAIC levels further underscores its close association with endothelial cell activation or injury. This supports the potential utility of t-PAIC as a biomarker for monitoring the progression of HTNV infection. Accumulating evidence indicates that immune-mediated mechanisms, characterized by heightened expression of inflammatory mediators, thrombocytopenia, and hematological disturbances—including coagulation dysfunction and enhanced fibrinolysis—constitute key elements in the pathogenesis of HFRS (Outinen et al., 2016; Laine et al., 2010). Furthermore, HFRS patients exhibit an increased risk of thrombotic complications, as evidenced by higher incidences of DIC, AMI, and stroke. Given the central role of t-PAIC in regulating human fibrinolytic activity (Suzuki et al., 2009; Tsantes et al., 2008; Chen et al., 2024), these pathophysiological processes likely contribute to the dynamic changes in t-PAIC levels and further highlight the potential value of t-PAIC in assessing disease severity and tracking clinical progression in HFRS patients. Of note, early studies have demonstrated that the onset of HFRS is associated with immune dysfunction and pathological damage resulting from dysregulation of the immune system (Huang et al., 1994). Furthermore, PAI-1 has been reported to function as a procoagulant, proinflammatory, and profibrotic molecule, and it also plays a pivotal role in innate immunity by modulating cell migration and phagocytosis (Yasar Yildiz et al., 2014; Agirbasli, 2005). Therefore, considering the pathophysiological features induced by HTNV infection—such as inflammatory conditions, immune dysfunction, and the upregulation of PAI-1 along with its interaction with t-PA leading to the formation of t-PAIC—it is likely that these factors collectively contribute to the overexpression of this complex. These further underscores the critical role of t-PAIC in the progression of HFRS.

Given that thrombocytopenia is a hallmark of HFRS, the pivotal role of t-PAIC in regulating fibrinolytic activity, and the close interplay between PLT and t-PAIC in thrombosis formation (Muranyi et al., 2005; Mehta and Shapiro, 2008), we investigated the correlation between t-PAIC and PLT. Our findings reveal an increasing trend in t-PAIC levels concurrent with the progression and worsening of HFRS, demonstrating a significant negative correlation between this complex and PLT count. As previously reported, the structural and functional properties of platelets contribute to their involvement in host defense against infection. It has been documented that agonist-induced platelet activation enhances interactions among platelets, humoral immune components, leukocytes, and endothelial cells (Yeaman, 1997). These interactions may lead to platelet adhesion and sequestration, resulting in reduced levels of circulating peripheral platelets during HTNV infection. These further underscores the negative association between t-PAIC and PLT, highlighting the potential significance of t-PAIC in the pathogenesis of HFRS. Likewise, acute kidney injury or failure represents another major clinical manifestation of HTNV infection. Renal function indices such as Cr, urea, and UA are critical markers reflecting renal impairment (Jonsson et al., 2010). Therefore, we also evaluated the relationships between t-PAIC and these biochemical parameters. Our data indicated that t-PAIC levels in HFRS patients are positively correlated with elevations in Cr, urea, and UA. Notably, both components of t-PAIC—t-PA and PAI-1—are primarily secreted by endothelial cells. Moreover, HTNV has been shown to infect human tubular epithelial cells, glomerular endothelial cells, and podocytes (Suzuki et al., 2009; Tsantes et al., 2008; Krautkramer et al., 2011), Consequently, elevated t-PAIC levels may indirectly reflect vascular endothelial dysfunction and renal tissue damage during the course of HFRS, suggesting its potential value as a biomarker for disease severity. Furthermore, as prior studies have documented that coagulation disorders can also occur in patients with HFRS, refractory shock, hemorrhage-related complications, DIC, and MODS are key contributors to mortality in severe HFRS cases (Jiang et al., 2016). Therefore, the correlation between t-PAIC levels and coagulation parameters, specifically APTT and PT, was analyzed. It was observed that the levels of these two indices increased progressively during the course of HFRS, which is consistent with findings from previous studies (Chen et al., 2024). Consequently, elevated t-PAIC levels were positively correlated with the increases in APTT and PT. Given that signs of DIC are commonly observed during acute hantavirus infection—manifested by prolonged APTT and PT, as well as decreased platelet counts (Sundberg et al., 2011; Lee, 1987)—the observed association between coagulation parameters and t-PAIC further suggested the potential role of this complex in the pathogenesis and progression of HFRS. On the other hand, a statistical significance in Cr, APTT, and PT was not found in the ordinal logistic regression analysis. Considering that the association of Cr with hyperuricemia in reflecting the renal status, and the relationship between reduction in ALB levels and prolongation of APTT and PT (Chihara et al., 2025; Kim et al., 1997; Mumford et al., 2000). Both of which further suggesting the collinearity of these parameters in statistical analysis and leading to the non-significant difference of these parameters in this study.

In addition, given that hypoalbuminemia is well recognized as being associated with complications and increased mortality in patients with acute infectious diseases and considering that this condition frequently occurs during the acute phase of HFRS (Carratala et al., 2003; Kim et al., 2003). The fluctuation tendency of ALB in varying severity of HFRS, and the relationship between this molecule and t-PAIC were investigated. Indeed, a decline in ALB levels was observed, along with an inverse correlation between ALB and t-PAIC. Since vascular leakage of serum proteins—attributable to increased vascular permeability, urinary protein loss, reduced intestinal protein absorption, and enhanced fibrinolysis—has been documented in hantavirus infection, these mechanisms likely act synergistically to contribute to the observed hypoalbuminemia (Laine et al., 2010; Kim et al., 2003). Notably, previous studies have reported significant impairments in the function, composition, and particle size of HDL during the oliguric phase of HFRS. Moreover, evidence indicates that HDL participates in lipopolysaccharide binding during bacterial infections and plays a role in protective immune responses against viral infections (Cho et al., 2008; Ramirez et al., 2004; McDonald et al., 2003). In light of these findings, we further explored the association between HDL and t-PAIC in this study. Our results demonstrate that changes in t-PAIC levels are negatively correlated with fluctuations in HDL concentrations. This relationship may be attributed not only to the intrinsic role of HDL in antiviral defense but also to the disruption of metabolic homeostasis, as elevated UA levels may promote structural and functional modifications of HDL (Kontush and Chapman, 2006).

The complexity and heterogeneity observed in the clinical course and progression of HFRS represent a significant challenge that warrants careful consideration. The identification of a reliable biomarker reflecting disease severity or progression would greatly enhance patient management and therapeutic decision-making. ROC analysis demonstrated that t-PAIC exhibits remarkable predictive value for assessing the severity of HFRS. Furthermore, conventional laboratory parameters—such as PLT count, Cr, urea, and UA—also show certain predictive capacity for disease severity. However, the clinical utility of these traditional markers is substantially limited by imbalances between sensitivity and specificity, as well as by preanalytical interferences such as erythrocyte fragments and hemolysis, which can compromise the accuracy of laboratory testing. Therefore, a combined approach incorporating t-PAIC with one or more conventional parameters appears to be an optimal strategy for clinical practice—a finding supported by the present study. As shown, the predictive performance improves progressively with the number of combined parameters. Notably, the combination of t-PAIC with PLT, Cr, urea, and UA yields an AUC equivalent to that of the comprehensive model including all parameters. Although the latter demonstrates higher sensitivity, this does not diminish the significant contribution of t-PAIC in predicting HFRS severity.

Although this study demonstrated the potential predictive value of t-PAIC for the severity and clinical course of HFRS, the inherent limitations of this study should also be acknowledged. Specifically, the study was conducted at a single center with a limited sample size; therefore, future investigations involving a larger cohort across multiple medical centers are warranted to further validate the present findings. Furthermore, as the study population encompasses multiple sequential phases—including patient admission, specimen collection, and laboratory analyses—standardization and harmonization of these procedures will be essential in subsequent research. Additionally, potential experimental and human errors, as well as the overall management efficiency concerning the study population, must be carefully addressed to ensure data reliability and study integrity.

## 5 Conclusions

Taken together, this study demonstrates that elevated plasma levels of t-PAIC in patients HFRS are significantly correlated with key clinical manifestations of the disease, including coagulation abnormalities and renal injury. These findings suggest that t-PAIC expression is closely associated with disease severity, a conclusion further supported by ROC curve analysis. Therefore, monitoring plasma t-PAIC levels—particularly in combination with multiple conventional clinical indices—may contribute to the assessment of disease progression and improve the clinical management and therapeutic evaluation of HFRS patients.

## Data Availability

The raw data supporting the conclusions of this article will be made available by the authors, without undue reservation.
